# Predictive Power of Long-Read Whole-Genome Sequencing for Rapid Diagnostics of Multidrug-Resistant Brachyspira hyodysenteriae Strains

**DOI:** 10.1128/spectrum.04123-22

**Published:** 2023-01-05

**Authors:** Nick Vereecke, Nadine Botteldoorn, Charlotte Brossé, Caroline Bonckaert, Hans Nauwynck, Freddy Haesebrouck, Filip Boyen, Dominiek Maes, Sebastiaan Theuns

**Affiliations:** a Laboratory of Virology, Faculty of Veterinary Medicine, Ghent University, Merelbeke, Belgium; b PathoSense BV, Lier, Belgium; c Animal Health Care Flanders, Lier, Belgium; d Department of Pathobiology, Pharmacology and Zoological Medicine, Faculty of Veterinary Medicine, Ghent University, Merelbeke, Belgium; e Unit of Porcine Health Management, Faculty of Veterinary Medicine, Ghent University, Merelbeke, Belgium; Texas A&M University

**Keywords:** nanopore, swine dysentery, infectious diseases, genomics, antimicrobials

## Abstract

Infections with Brachyspira hyodysenteriae, the etiological agent of swine dysentery, result in major economic losses in the pig industry worldwide. Even though microbial differentiation of various *Brachyspira* species can be obtained via PCR, no quick diagnostics for antimicrobial susceptibility testing are in place, which is mainly due to the time-consuming (4 to 7 days) anaerobic growth requirements of these organisms. Veterinarians often rely on a clinical diagnosis for initiating antimicrobial treatment. These treatments are not always effective, which may be due to high levels of acquired resistance in B. hyodysenteriae field isolates. By using long-read-only whole-genome sequencing and a custom-trained Bonito base-calling model, 81 complete B. hyodysenteriae genomes with median Q51 scores and 99% completeness were obtained from 86 field strains. This allowed the assessment of the predictive potential of genetic markers in relation to the observed acquired resistance phenotypes obtained via agar dilution susceptibility testing. Multidrug resistance was observed in 77% and 21% of the tested strains based on epidemiological cutoff and clinical breakpoint values, respectively. The predictive power of genetic hallmarks (genes and/or gene mutations) for antimicrobial susceptibility testing was promising. Sensitivity and specificity for tiamulin [*tva*(A) and 50SL3^N148S^, 99% and 67%], valnemulin [*tva*(A), 97% and 92%), lincomycin (23S^A2153T/G^ and *lnuC*, 94% and 100%), tylvalosin (23S^A2153T/G^, 99% and 93%), and doxycycline (16S^G1026C^, 93% and 87%) were determined. The predictive power of these genetic hallmarks is promising for use in sequencing-based workflows to speed up swine dysentery diagnostics in veterinary medicine and determine proper antimicrobial use.

**IMPORTANCE** Diagnostics for swine dysentery rely on the identification of *Brachyspira* species using molecular techniques. Nevertheless, no quick diagnostic tools are available for antimicrobial susceptibility testing due to extended growth requirements (7 to 14 days). To enable practitioners to tailor antimicrobial treatment to specific strains, long-read sequencing-based methods are expected to lead to rapid methods in the future. Nevertheless, their potential implementation should be validated extensively. This mainly implies assessing sequencing accuracy and the predictive power of genetic hallmarks in relation to their observed (multi)resistance phenotypes.

## INTRODUCTION

Brachyspira hyodysenteriae is the major etiological agent of swine dysentery (SD) in pigs (1). However, in recent years, Brachyspira hampsonii and B. suanatina have also been linked to SD (2, 3). This spirochete bacterium mainly causes disease in grower or finisher pigs by targeting the large intestine, causing a severe mucohemorrhagic colitis (4). While disease outcome varies from dehydration to weight loss and in some cases death, it contributes to significant economic losses in the swine industry worldwide. This loss is mainly due to decreased feed conversion, uneven growth, treatment costs, and increased mortality (4–6).

Eradication of B. hyodysenteriae from pig farms is possible, but the success rate can be low and reinfections may occur (7). So far, there are no efficient commercial vaccines, and the use and efficacy of autogenous vaccines is poorly documented (8–10). First-line treatment consists of antimicrobial agents, including pleuromutilins (tiamulin and valnemulin) as the first choice, followed by macrolides (tylvalosin) and lincosamides (lincomycin), possibly in combination with tetracyclines (doxycycline) (7; antimicrobial consumption and resistance in animals (AMCRA) guidelines, 2022). Unfortunately, reduced susceptibility of B. hyodysenteriae to various antimicrobials has been reported in recent years. While almost 100% acquired resistance is seen against the macrolide tylosin, various reports show increased acquired (multi)drug resistance against all aforementioned antimicrobials (11; AMCRA guidelines, 2022). Thus, proper monitoring of (multi)drug resistance in B. hyodysenteriae strains is of utmost importance to study and understand its epidemiological context. Nevertheless, antimicrobial susceptibility testing (AST) for *Brachyspira* spp. is cumbersome due to its anaerobic growth and its requirement for extended incubation time (up to 7 to 14 days) prior to identification and potential AST against relevant antibiotics (12, 13).

While the agar dilution method has been used extensively to measure MICs for *Brachyspira* spp., a more standardized method using VetMIC-Brachy plates was developed (14). Nevertheless, many veterinary diagnostic laboratories involved in SD diagnostics do not yet employ this approach. Even though the broth microdilution approach was shown to be more accurate, its success still depends on effective growth and interpretation of acquired resistance based on reliable epidemiological cutoffs (ECOFFs) or clinical breakpoints (CBs) (15, 16). To circumvent this, molecular approaches might be advantageous in AST for *Brachyspira* spp. Whole-genome sequencing (WGS) has become affordable and broadly applicable in (metagenomic) diagnostics and monitoring of infectious diseases in veterinary and human medicine (17). While short-read sequencing has some limitations when complex bacterial genomes (e.g., peculiar GC content and genomic repeats) are analyzed, long-read alternatives such as Oxford Nanopore Technologies (ONT) nanopore-based sequencing have been shown to have great value in this context (18–22). Nevertheless, prior to its implementation in all-in-one diagnostics for identification, typing, and genetic AST, in-depth validation is required. This implies addressing raw read and consensus genome accuracy, but also linking virulence and antimicrobial resistance (AMR) phenotypes to specific genotypes and associated genetic markers. For example, a point mutation in the 16S rRNA gene (G1058C [Escherichia coli numbering]) has been linked to tetracycline resistance, and multiple mutations in the fifth domain of the 23S rRNA gene (peptidyl transferase loop; A2058G/T, T2528C, and G2535A/C) and 50S ribosmal protein L3 (RPLC) protein (N148S) have been shown to result in acquired resistance to macrolide, pleuromutilin, and lincosamide antibiotics in B. hyodysenteriae (23). Also, the *tva*(A) and transposon-linked *lnu*(C) genes have been identified as genetic factors in pleuromutilin and lincomycin resistance, respectively (1, 11, 24, 25).

This study sheds new light on the use of long-read nanopore WGS of Belgian B. hyodysenteriae strains by linking known and potential new genetic markers with AMR phenotypes against relevant antimicrobials. These markers can be potentially exploited in quick all-in-one diagnostic tools for SD to determine proper use of these drugs in the future. In addition, the genomes provided the opportunity to study Belgian epidemiology of B. hyodysenteriae between 2018 and 2020. To date, only combined data on phenotypes and genotypes for B. hyodysenteriae field strains have been published for strains collected before 2015 in some European countries, including Belgium (24), Switzerland (26), and the United Kingdom (UK) (27).

## RESULTS

### Generation of high-quality and complete B. hyodysenteriae genomes.

Genome assemblies were obtained from long-read-only data. Due to the lower assembly accuracy of conventional base-calling algorithms (default Guppy; median quality [Q] score of 36.2 ± 1.2) for nonstandard organisms, a custom-trained Bonito base-calling model was used to elevate raw read and consensus assembly accuracies. This allowed the generation of B. hyodysenteriae genomes with a median Q score of 51.1 (± 1.6) score based on eight independently sequenced B78 type strain controls (see Table S2 in the supplemental material). This is comparable to the B78 assembly obtained from a short-read-only assembled genome (Q50). Also, the average genome completeness was assessed (99.3% ± 0.3%), which was the same as that of the NCBI ATCC 27164 (B78) type strain reference (99.1%). A total of 81 (out of 86) complete and high-quality genomes were retained for subsequent analyses. Three genome assemblies showed doubtful classifications and suggested a potential mixture of B. hyodysenteriae with B. innocens and B. murdochii. Two other genomes showed lower genome completeness due to limited sequencing coverage because of substantial contamination of Desulfovibrio fairfieldensis. Therefore, these genomes were excluded from downstream analyses. Final B. hyodysenteriae genome assemblies showed a median GC content of 27.1% (± 0.0%) and 395 (± 23) predicted genes (versus 27.0% GC and 385 predicted genes for the NCBI B78 type strain). All sequences, their accession numbers, and quality measures are summarized in Table S1.

### Distribution and occurrence of various sequence types in Belgian pigs.

**FIG 1:**
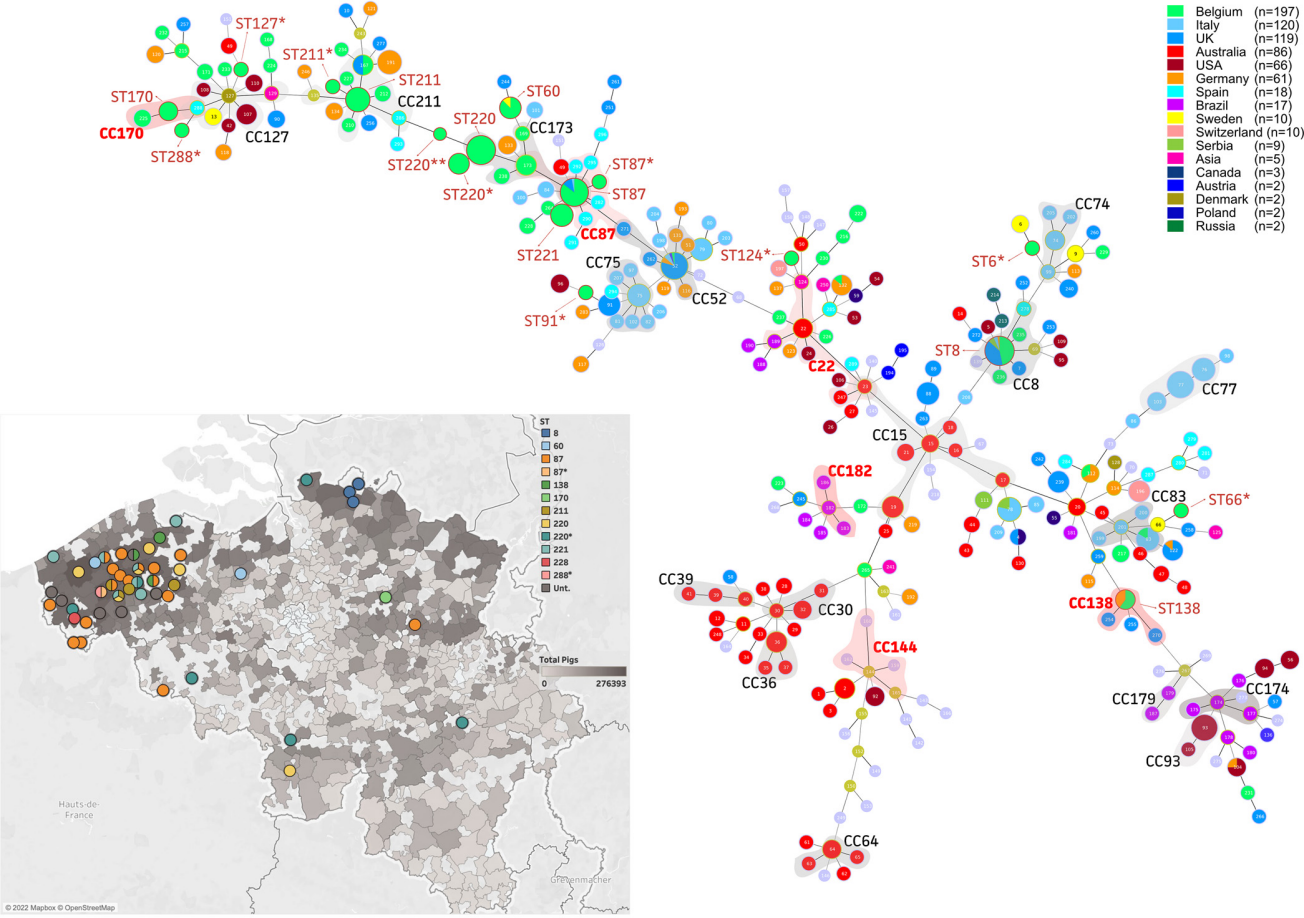
Presence and distribution of B. hyodysenteriae STs in Belgium. Full goeBURST MLST profile of all available MLST profiles (*n* = 729). All Belgian STs are shown in light green, and relevant STs of the new Belgian data sets are encircled in dark red, with new STs indicated with an asterisk. CCs previously identified by Joerling et al. (35) are highlighted in gray, and new CCs with more than two STs are highlighted in red. The color key includes the total number of available strains. (Inset) MLST distribution in Belgium, indicating the total pig population per city (gray background). The color key shows STs. If more than one ST was identified in a farm, multiple colors are present within the circle. Potential new STs are indicated with asterisks and untypeable strains (Unt.) with dark gray circles. The map was created with the licensed software Tableau (77).

All genomes were subjected to multilocus sequence typing (MLST), which showed the existence of at least 12 known B. hyodysenteriae sequence types in Belgium. As shown in Fig. 1, most Belgian sequence types (STs) clustered together in the goeBURST analysis, showing the linkage between ST60, ST87, ST220, ST211, and ST170. ST87 and ST170 were shown to be founders of two new clonal complexes (CCs), named CC87 and CC170, respectively. While some strains were untypeable after analysis against the PubMLST databases, the goeBURST analysis suggested the closest relationship with existing STs, including ST6* (*n* = 1; *alp* allele 7 to 11), ST66* (*n* = 1; *alp* allele 11 to 7), ST91* (*n* = 1; *glpK* allele 24 to 7 and *thi* allele 6 to 13), ST124* (*n* = 1; *alp* allele 8 to 18 and *glpK*^T447G^), ST127* (*n* = 1; *adh* allele 11 to 13, *gdh* allele 5 to 17, and *glpK* allele 6 to 4), and ST211* (*n* = 1; *est* allele 28 to 17 and *thi* allele 21 to 3). When we focused on our Belgian STs, the most dominant sequence types were ST87 and ST220, at 35% (28/81) and 14% (11/81), respectively (Fig. 1). Both STs showed a close relationship to other STs, due to the presence of a single mutation or differential allele in a single MLST gene. These closely related STs were designated ST87* (*n* = 1; *glpK*^T852C^), ST220* (*n* = 3; *pgm*^C508T^), and ST220** (*n* = 4; *gdh* allele 6 to 1). Since they differ in only 1 allele, they all belonged to their respective CC. While most STs were identified in different swine herds, some were detected only at a specific geographical location in Belgium. As shown in Fig. 1, ST8 (dark blue; *n* = 5), ST170 (light green; *n* = 1), and ST228 (red; *n* = 1) and ST288* (pink; *n* = 1; *est* allele 8 to 17) were identified only in the provinces of Antwerp, Limburg, and West Flanders, respectively.

### Increased multidrug resistance in Belgian B. hyodysenteriae strains.

The distribution of MICs for tested antimicrobial compounds for all B. hyodysenteriae strains (*n* = 81) is shown in Table 1 and Table S4. While 77.8% and 80.2% of new Belgian strains showed MICs above the ECOFF for tiamulin and valnemulin, respectively, MICs for 44.4% of all new strains were above the tiamulin-specific CB (no CB value is available for valnemulin with the current testing methodology). For lincomycin and tylvalosin, 92.5% and 90.1% of all new Belgian strains showed MICs above their corresponding ECOFFs. In comparison, 63.8% and 61.7% of strains had MICs above the CB, suggesting potential nonresponders to them. Finally, for doxycycline, 86.4% and 42% of our new strains showed MICs above the ECOFF and CB, respectively. The MIC_50_s and MIC_90_s for each antimicrobial compound were also calculated (Table 1).

**TABLE 1:** Distribution of MICs of all 81 studied B. hyodysenteriae strains^*a*^

MIC (μg/mL)	0.03125	0.0625	0.125	0.25	0.5	1	2	4	8	16	32	64	128	256
Tiamulin	6	1	5	6 |	5	10	**12** |	3	5	8	20			
Valnemulin	13	3 |	1	1	4	1	11	**10**	7	7	23			
Lincomycin			3	1	1	1 |	2	2	6	13 |	**20**	15	16	
Tylvalosin			1	1	3	3 |	7	1	2	13 |	8	**14**	19	9
Doxycycline			1	6	3 |	9	12	**15** |	12	5	17			

a Overview of MICs obtained for the most relevant antimicrobial classes, including pleuromutilins (tiamulin and valnemulin), lincosamides (lincomycin), macrolides (tylvalosin), and tetracyclines (doxycycline). The first and second vertical line in each row indicate the ECOFF and CB, respectively, for that drug, as summarized by Stubberfield et al. (1). For valnemulin, no CB is available. Boldface indicates MIC_50_s; underlining indicates MIC_90_s, which are higher than the maximal measured antibiotic concentration. One sample lacked phenotypic information for both lincomycin and doxycycline. Number of isolates indicated in the grey zones, represent strains for which MIC values were lower (<) or higher (>) than the lowest and highest tested antimicrobial concentration, respectively.

**FIG 2:**
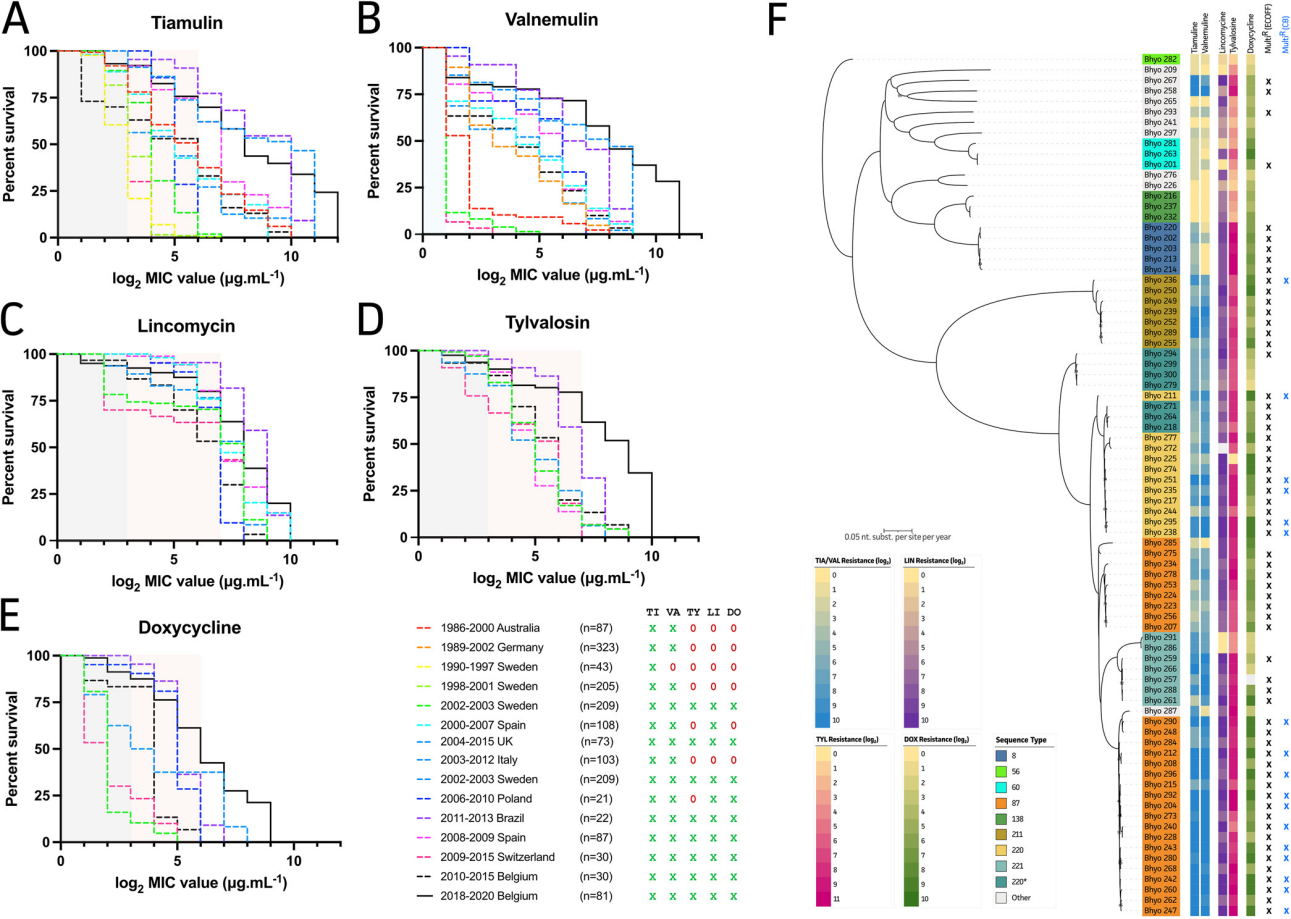
Survival curves and multidrug resistance indicated by B. hyodysenteriae MICs for all tested antimicrobials. (A to E) Available data from different periods and countries are presented with differential colors (key). This includes data from Australia (38), Germany (13), Sweden (15), Spain (23, 39), the UK (1, 11), Italy (41), Poland (40), Brazil (42), and Switzerland (26). Belgian strains from 2010 to 2015 (24) and 2018 to 2020 (this study) are indicated with dashed and solid black lines, respectively. The availability and lack of availability of antimicrobial resistance data for the antimicrobial drugs of interest are shown with a green X and red 0, respectively. Blue and red represent wild-type strains according to the ECOFF and CB, as summarized by Stubberfield et al. (1). (F) Rooted ML tree of new Belgian isolates with their antimicrobial resistance profiles and suggested multidrug resistance based on the ECOFF (black X) or CB (blue X).

In a MIC survival analysis to compare MICs for our new strains to all available B. hyodysenteriae MICs, a clear right-handed shift was observed. This was also the case for MICs of all tested antimicrobials compared to MICs for Belgian isolates from 2010 to 2015 (24) (Fig. 2, dashed versus solid black line). In addition, high levels of acquired resistance against lincomycin and tylvalosin had been observed in all countries as early of the 2000s (Fig. 2C and D). In the case of valnemulin, B. hyodysenteriae strains from Switzerland (Fig. 2B, pink), Sweden (green), and Australia (red) showed less acquired resistance than those from all other countries. The same trends could be observed in Switzerland and Sweden with regard to acquired resistance against doxycycline (Fig. 2E; no data available for Australia). Conversely, isolates from Brazil (Fig. 2B, purple), Spain (light blue and dark pink), and our new strains (solid black line) collocated with the highest acquired resistance levels for all tested antimicrobials. Importantly 76.5% or 21.0% of currently tested Belgian strains (based on ECOFFs or CBs, respectively) were classified as multidrug resistant, as they showed acquired resistance to one pleuromutilin (tiamulin or valnemulin), doxycycline, and one of the macrolide-lincosamide-streptogramin B (MLSB) antibiotics (lincomycin or tylvalosin) (Fig. 2F). It is noteworthy that the most abundant STs (ST87 and ST220) in Belgium showed 96.4% and 100% multidrug resistance, respectively, when the ECOFF was used. Still, 39.3% (11/28) and 45.5% (5/11) of ST87 and ST220 strains, respectively, remained classified as multidrug resistant when CB values were used. Overall, 62.2% (52/81) or 17.3% (14/81) of our new strains showed acquired resistance to all four tested antimicrobial classes, based on ECOFF and CB, respectively.

### Identification of genetic hallmarks for B. hyodysenteriae genotypic AST.

**FIG 3:**
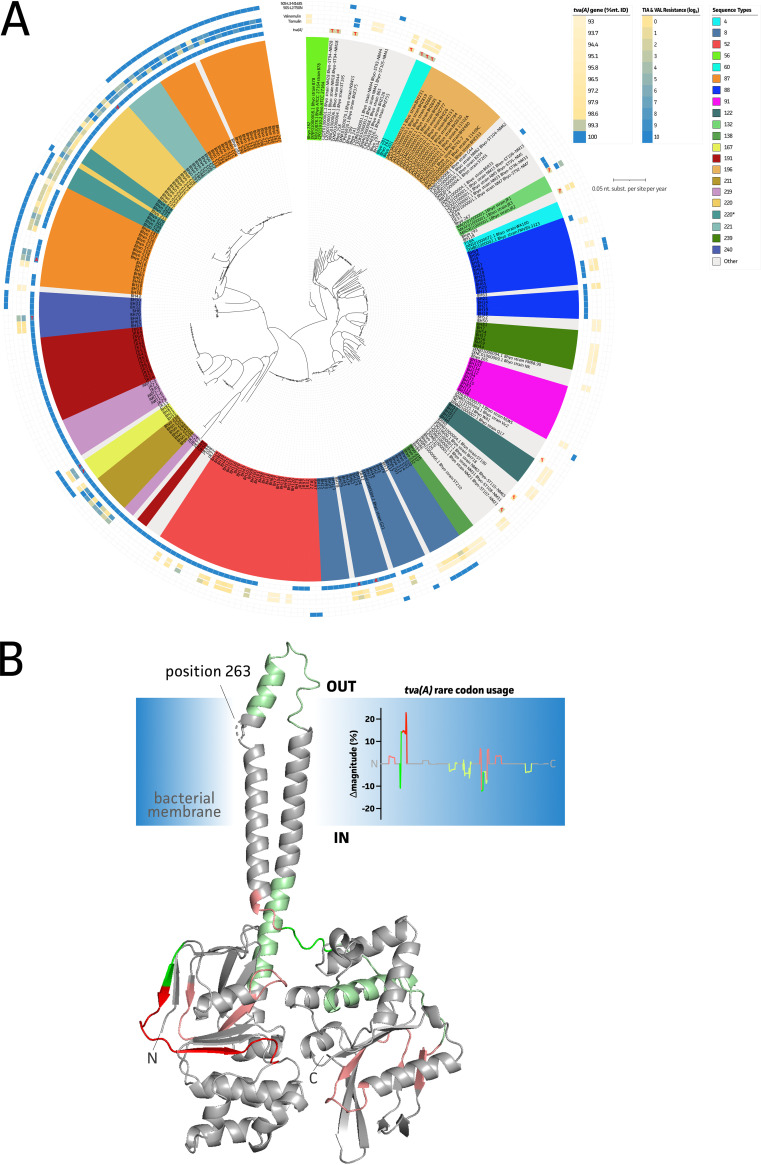
Identification of genetic hallmarks for pleuromutilin (tiamulin and valnemulin) resistance in B. hyodysenteriae. (A) Overview of all available WGS data from B. hyodysenteriae strains in relation to their phenotypes against tiamulin and valnemulin. Strains are colored according to their STs. When fewer than 3 strains belonged to a group, they were grouped as “other.” The presence of genetic markers and phenotypic resistance profiles are shown on outer circles. From inside to outside, circles represent the presence and nucleotide identity of the *tva*(A) gene and an A263T mutation highlighted in red, phenotypic resistance profiles of tiamulin and valnemulin, and genetic markers in the 50S ribosomal protein L2 (T50N) (11) and L3 (N148S) (54), respectively. (B) Alphafold2 protein structure of the *tva*(A) protein, showing a typical ABC exporter structure (28). Hypothetical embedment in the bacterial membrane is shown in blue; positions with differential codon usage are highlighted in green and red when more common or rare codons are used, respectively. The latter was obtained by comparing mutated *tva*(A) genes to the reference sequence (LT970863).

Since a high level of acquired antimicrobial (multi)drug resistance was observed in our new B. hyodysenteriae strains, a genotypic screening was performed to identify known resistance markers. All available B. hyodysenteriae genomes and associated AMR phenotypes were used to supplement our analyses. As shown in Fig. 3A, the *tva*(A) gene is widely distributed among the B. hyodysenteriae population. Its presence showed sensitivity and specificity (Sn/Sp) of 94.4%/61.8% and 99.1%/84.6%, respectively, for tiamulin (MIC, >0.25 μg mL^−1^ for Belgian and UK strains) and valnemulin (MICs, >0.50 μg mL^−1^ and >0.25 μg mL^−1^ for Belgian and UK strains, respectively) (Tables S3 and S4). Figure 3B shows the modeled structure of the *tva*(A) protein, as obtained by Alphafold2. This showed a clear homology to known ABC exporter proteins (28). In some strains, a mutated *tva*(A) gene was identified (93.81% to 99.87% nucleotide identity compared to the reference sequence [GenBank no. LT970863]). These mutations resulted in only a single amino acid alteration at position 263 (Ala to Thr) in most of the mutated strains. For the other strains, the observed mutations were classified as synonymous; however, no correlation with lowered pleuromutilin resistance phenotypes could be drawn (Fig. 3A, red letters). Most of the *tva*(A)-positive and pleuromutilin-susceptible strains harbored silent mutations in the *tva*(A) gene, resulting in differential use of codons. The use of more common codons might impact proper protein folding. These regions are highlighted on the *tva*(A) protein structure and associated difference plot (Fig. 3B). A GAG, ATT, and ATT codon were present at positions 74, 76, and 77 of the *tva*(A) gene, respectively, compared to GAA, ATC, and AAC in the resistant strains. Assuming that these potentially faulty proteins prevent proper functionality of the ABC exporter or its potential role in ribosomal protection, the Sn/Sp measures changed to 92.6%/69.1% and 97.3%/92.3% for tiamulin and valnemulin, respectively. Some of the Belgian strains tested here (5/81) showed elevated MICs of tiamulin without harboring the *tva*(A) gene. Five of these *tva*(A)-positive but pleuromutilin-susceptible strains belonged to the ST8 group and showed an apparent correlation with the N148S mutation in the 50S ribosomal protein subunit L3. Adding this genetic hallmark to assess predictivity of tiamulin and valnemulin resistance further increased the Sn to 99.1% and 100%, respectively. A slightly lower Sp (69.1% to 66.7%) was observed for tiamulin, whereas a greater reduction in valnemulin Sp (92.3% to 71.2%) was observed. Finally, a T50N mutation in the 50S ribosomal protein subunit L2 showed no overall correlation with pleuromutilin resistance phenotypes.

**FIG 4:**
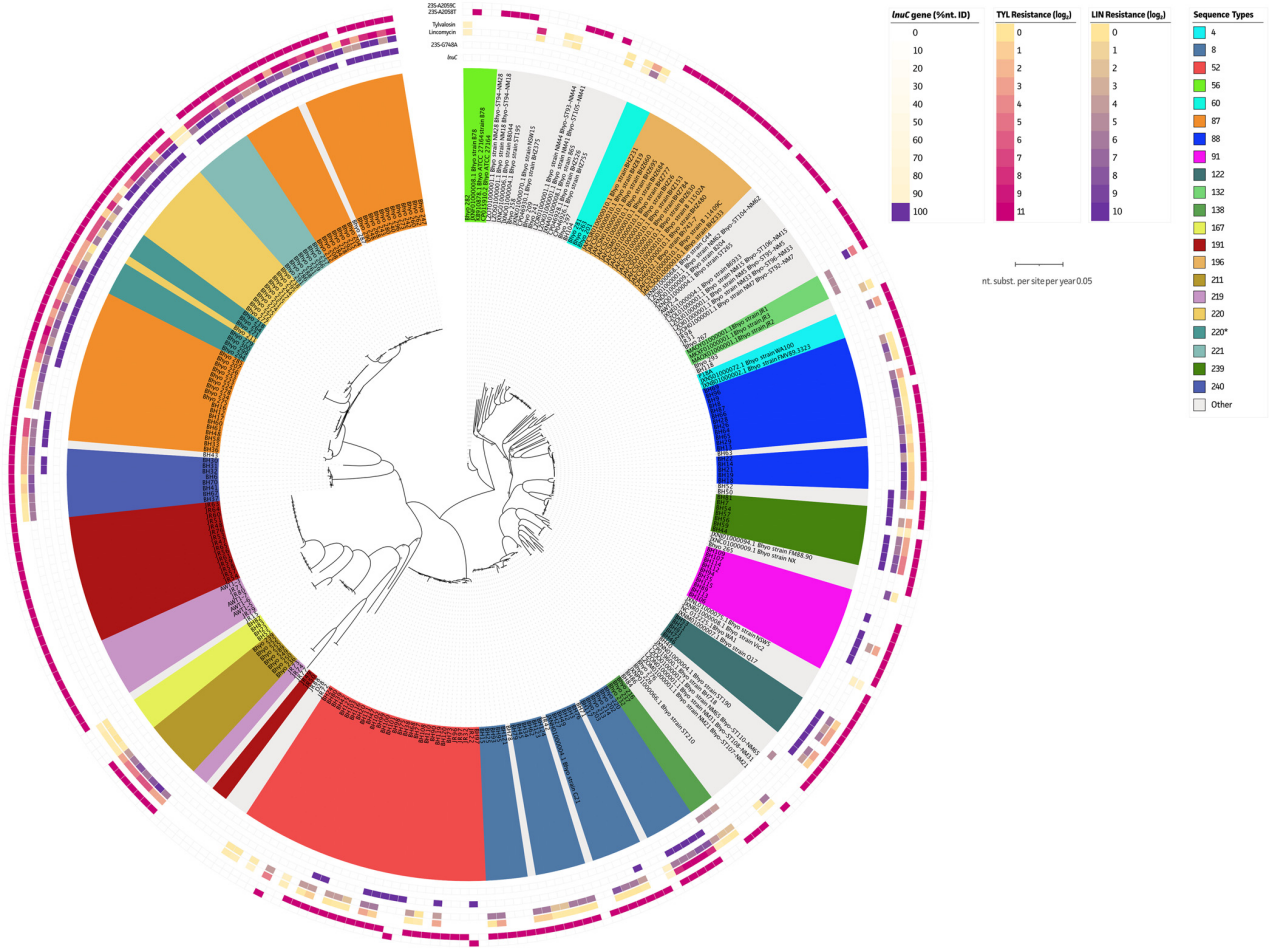
Identification of genetic hallmarks for lincosamide (lincomycin) and macrolide (tylvalosin) resistance in B. hyodysenteriae. Overview of all available WGS data from B. hyodysenteriae strains in relation to their phenotypes against lincomycin and tylvalosin. Strains are colored according to their STs. Whenever fewer than 3 strains belonged to a group, they were grouped as “other.” The presence of genetic markers and phenotypic resistance profiles is shown on the outer circles. From inside to outside, circles represent the presence and nucleotide identity of the *lnuC* gene, lincomycin-associated 23S rRNA gene mutation (G484A), phenotypic resistance profiles of lincomycin and tylvalosin, and genetic markers in the 23S rRNA gene (A2153T/G and A2154G) (1, 48).

Acquired resistance to lincomycin and tylvalosin showed comparable resistance phenotypes, suggesting a cross-resistance mechanism (29). A conversion of G to A at position 846 (position 748 in the E. coli numbering) of the 23S rRNA gene could explain only part of the observed acquired lincomycin resistance phenotypes (Fig. 4 and Fig. S1A). A better association was shown for the A2153T/G genotype (position 2058 in the E. coli numbering). This genetic hallmark showed Sn/Sp of 91.8%/100% and 98.5%/92.9% for lincomycin (MICs, >2 μg mL^−1^ and >1 μg mL^−1^ for Belgian and UK strains, respectively) and tylvalosin (MICs, >4 μg mL^−1^ and >1 μg mL^−1^ for Belgian and UK strains, respectively), respectively (Tables S3 and S4). Interestingly, the *lnuC* gene could explain lincomycin resistance of three additional Belgian strains that did not harbor the aforementioned 23S rRNA gene mutation, increasing the lincomycin Sn to 93.8% and maintaining a 100% Sp (Tables S3 and S4). These strains showed slightly lower lincomycin MICs (8 to 16 μg mL^−1^) compared to >32 μg mL^−1^ with the A2153T/G (position 2058 per E. coli numbering) mutation. The *lnuC* gene was specific to some STs, being identified in ST132 (Germany) and ST138 (Belgium), although no phenotypic data were available for the German strains. The UK data set also showed 2 strains with the same A2154G (position 2059 per E. coli numbering) instead of the A2153T/G genotype in the 23S rRNA gene. However, it has not been associated with resistance so far. Due to its localization in the domain V loop of the 23S rRNA gene, this suggests an additional genetic hallmark that might explain acquired resistance to lincomycin and tylvalosin (Fig. 2A and B).

**FIG 5:**
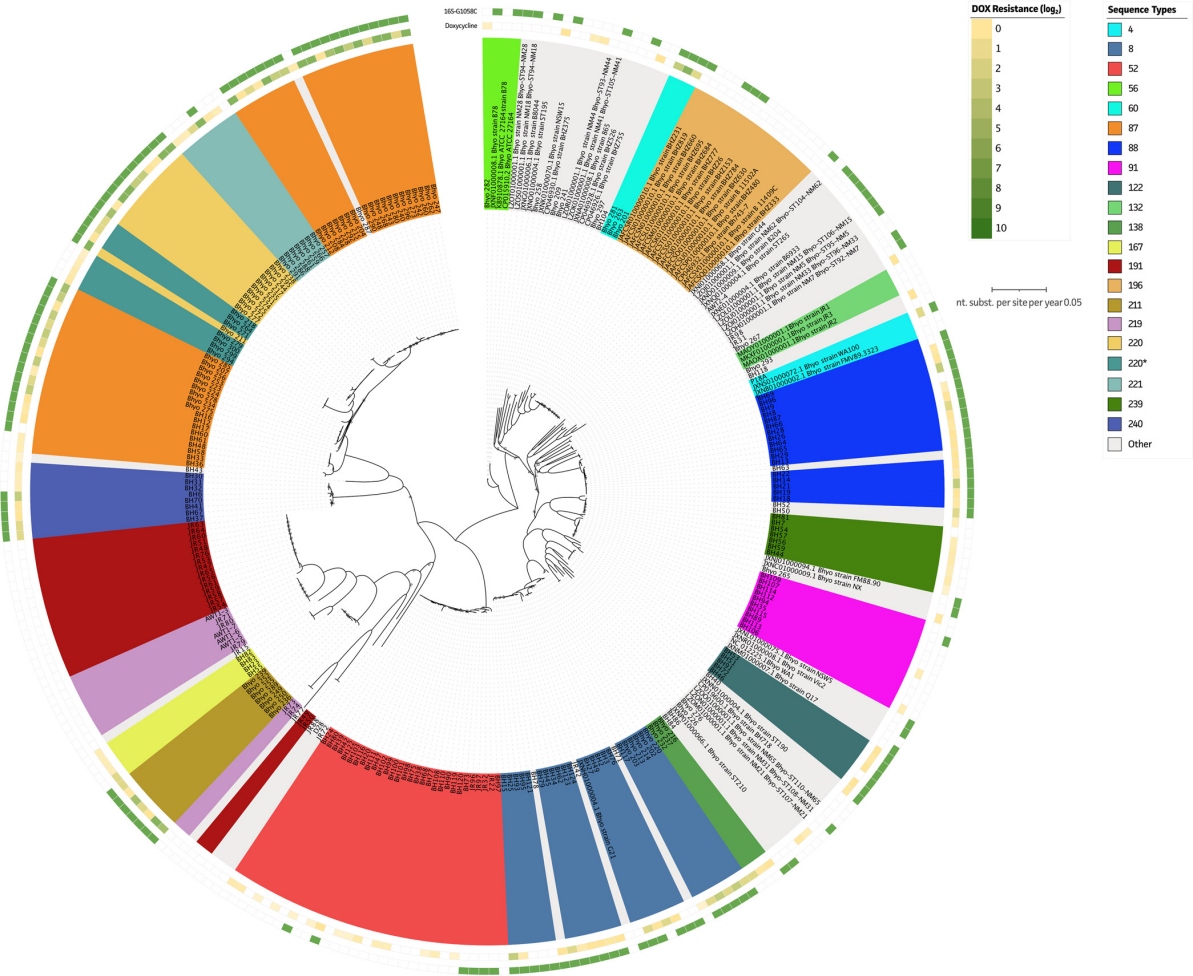
Identification of genetic hallmarks for tetracycline (doxycycline) resistance in B. hyodysenteriae. Overview of all available WGS data from B. hyodysenteriae strains in relation to their phenotypes against doxycycline. Strains are colored according to their STs. Whenever fewer than 3 strains belonged to a group, they were grouped as “other.” The presence of genetic markers and phenotypic resistance profiles is shown on outer circles. From inside to outside, circles represent the phenotypic resistance profile of doxycycline, followed by genetic markers in the 16S rRNA gene (G1026C/T) (78).

Finally, doxycycline phenotypes were verified for mutations on the 16S rRNA gene target (Fig. 5). The G1026C (position 1058 per E. coli numbering) mutation showed a clear correlation with doxycycline resistance and showed an Sn and Sp of 93% and 87% for acquired resistance to doxycycline (MICs, >1 μg mL^−1^ and > 0.5 μg mL^−1^ for Belgian and UK strains, respectively) (Table S3). Even though three potentially new 16S rRNA gene mutations were identified in the vicinity of the doxycycline drug-binding region (T957C, G1155A, and C1180T), none of them showed a clear correlation with the observed phenotypes (Fig. S1B). Most of these were associated solely with specific STs (e.g., ST221 and ST87).

## DISCUSSION

Our study investigated long-read nanopore-based sequencing as a new tool to obtain genetic AST in SD diagnostics. This work was initiated because an increased number of PCR-positive B. hyodysenteriae cases were seen at Animal Health Care Flanders since 2015 (12.6% to 20.4% in 2018). While standard antimicrobial susceptibility testing for widely used antimicrobials, such as pleuromutilins (tiamulin and valnemulin), macrolides (tylvalosin), lincosamides (lincomycin), and tetracyclines (doxycycline), was performed using the agar microdilution method, 81 complete and high-quality genomes were also generated. Combining genomic and phenotypic data allowed us to identify known and potential new genetic markers and asses their predictive power (Sn and Sp) for future use in quick genetic AST. Our study also shed light on Belgian B. hyodysenteriae population epidemiology between 2018 and 2020. This allowed us to address population diversity and assess the relationship of our strains to those available from other countries.

Our work showed the use and value of whole-genome sequencing using long-read-only nanopore (ONT) reads. While training of the Bonito base-caller was an elaborate bioinformatics task for early releases of the software, recent software releases (from v.0.4.0 onward) have allowed more user-friendly base-calling training, as it is completely embedded within the Bonito software. Here again, we have demonstrated the use of a custom-trained Bonito base-caller model in the generation of complete and high-quality genome assemblies for nonstandard bacterial species (21, 30). Even though ONT’s raw read accuracy has reached a Q20 level upon the implementation of new pore and motor protein chemistries at the time of writing, these accuracy improvements account only for bacterial species for which the default guppy base-caller model has been trained (communication ONT, London Calling 2022). This was previously demonstrated for Mycoplasma bovis, which also shows a diverging genomic buildup with a 29% GC content compared to 27% in B. hyodysenteriae (21, 31). Since native DNA is sequenced, DNA modifications, such as methylations, will also affect the efficiency of converting k-mer squiggles to actual base calls. Thus, the use of long-read-only data was reflected in the generation of above-Q50-level consensus genomes. While this is comparable to genome assemblies obtained from short-read alternatives, the additional benefit is the generation of complete circular B. hyodysenteriae genomes and plasmids and the potential to study its associated methylome (21, 32–34).

The whole-genome sequences were used to study strain diversity and distribution in Belgian pig herds suffering from clinical signs resembling SD. A minimum of 12 different STs were identified, showing major dominance of ST87 (35%) and ST220 (14%) in Belgium. Also, some closely related STs (ST87*, ST220*, and ST220**) were identified that deviated from these dominating STs. As described by Stubberfield et al., ST8 and ST87 were already circulating in Europe before 2018 and are thus potentially exchanged between these countries through pig transport (1). While ST8, ST60, ST87, ST112, and ST132 were reported to be circulating in Belgium between 2010 and 2018, only ST8, ST60, ST87, and ST228 showed a persistent presence in Belgium (7, 24). Notably, these studies comprised only 30 and 6 strains, respectively. While most STs occurred in different herds in Belgium, some were limited to a single geographic location (e.g., ST8 in Antwerp), suggesting different (inter)national contact/transport in these herds.

Our results are based on B. hyodysenteriae isolates that were obtained from many different Belgian pig farms suffering from clinical signs resembling SD. In this sense, they likely provide a good picture of the situation in such farms, but as the farms were not randomly selected, extrapolation should be done with care. Because of the SD problems, such farms might have used more antimicrobials to control SD than other farms, and therefore, the results might be an overestimation of the resistance problems in Belgium (Fig. 1). To the best of our knowledge, our results represent the most complete MLST analysis available to date. While previously described CCs were present in our analysis, our work showed the existence of at least 6 new CCs with more than 3 strains, of which two (CC87 and CC170) show a predominance of Belgian isolates (35). Of the 81 strains, seven did not allow proper determination of known STs. The untypeable character of these strains could be resolved through identifying their closest related STs via the goeBURST MST analysis. Most of these strains are spread across the MLST tree, supporting the hypothesis of various introductions of B. hyodysenteriae in Belgium.

These strains might also be a result of evolutionary pressure due to the extensive use of various antimicrobials in the context of eradication programs over the last years (7). Interestingly, these strains showed lower or no resistance and are thought to be newly emerging strains which, as such, have not been extensively exposed to antimicrobials yet. With increasing availability of WGS data, it is becoming clear that B. hyodysenteriae (and *Brachyspira* spp. in general) represents a genetically diverse population (1). This genetic diversity raises the hypothesis that B. hyodysenteriae as a species might represent pathogenic and less pathogenic genotypes, as described for other bacterial species, such as Streptococcus suis (36). Moreover, none of these strains clustered within a previously described weakly hemolytic German clade (Fig. 2 to 4) (37). As such, current data are thought to reflect only a small fraction of the actual diversity within the species B. hyodysenteriae. Even though new STs are introduced and emerge, their low levels of acquired resistance to various antimicrobials represent a ray of light in combating SD. Hence, thoughtful use of appropriate antimicrobial drugs will remain important to prevent the introduction of acquired resistance within these new STs. Again, this highlights the importance of faster AST in SD cases to determine proper antimicrobial treatment. This AST might also be very helpful when considering eradication programs and determining which type of eradication strategy might be most successful (7). Eradication strategies relying on antimicrobial treatment might not be preferred in case the pigs are infected with (different) multiresistant B. hyodysenteriae strains.

The phenotypic data from this study demonstrated the intense circulation of multidrug-resistant B. hyodysenteriae strains in Belgium. We also supplemented our results with an extensive literature review to picture the evolution of MICs over time in different countries. Notably, while our MICs were determined using the agar dilution technique, all other studies cited here used broth microdilution, as initially described by Karlsson et al. and later optimized by the use of VetMIC-Brachy plates (11, 38). This should be considered a pitfall of the current study, as phenotypic characterization of our strains was performed at a routine laboratory where the agar dilution method is currently applied for B. hyodysenteriae AST. Hence, it is important to emphasize that *Brachyspira* AST should be standardized in routine veterinary diagnostic laboratories all over the globe and not solely in academic research laboratories. Still, our comparison highlights important trends in acquired resistance in B. hyodysenteriae against the most used antimicrobials against SD. High levels of acquired resistance to lincomycin and tylvalosin have been observed in all countries where this has ben studied since the 2000s. Therefore, the use of these antimicrobials to treat B. hyodysenteriae should be discouraged (1, 11, 15, 23, 24, 26, 39–42).

Furthermore, it should be noted that the clinical effect of treatment may not necessarily correspond with susceptibility testing results (43). Interestingly, Australia, Sweden, and Switzerland show significantly less acquired resistance to valnemulin and doxycycline, which might be a result of different regulations regarding the use of antimicrobials (15, 26, 38). Alternatively, those data covered strains only up to 2000, 2003, and 2015 and therefore might not reflect the current state of acquired resistance in B. hyodysenteriae. More importantly, the MICs for all tested antimicrobials of the isolates in current study were higher than those for isolates from other countries. These were in line with MICs observed in isolates from Brazil and Spain (23, 42). While 1-log_2_ differences are to be expected when different MIC determination methods are used (44), the observed trends are worrying for Belgian pig herds with persistent SD problems. Of all currently tested Belgian strains, circulating between 2018 and 2020, 76.5% or 21.0% showed multidrug resistance phenotypes, based on ECOFF or CB, respectively. Moreover, 17.3% of our strains were considered to no longer respond to any of the approved antimicrobial drugs for SD (AMCRA guidelines, 2022).

To date, diagnosis of B. hyodysenteriae and differentiation from other *Brachyspira* spp. is restricted to identification using PCR. The implementation of AST is often not facilitated due to the extensive growth requirements of members the genus *Brachyspira* (45), forcing practitioners to rely on empirical treatments. Therefore, implementation of molecular sequencing-driven diagnostics could facilitate a quicker and cost-efficient all-in-one diagnostics approach (18, 22, 46). To implement genetic AST in diagnostics, a clear link and predictive power between resistance phenotypes and genotypes is required. Here, we show the clear relation and predictive power of known genetic hallmarks to be used in genetic AST. This allowed us to identify markers which can be exploited in innovative genomic diagnostics platforms. In the case of pleuromutilin resistance, using both the *tva*(A) gene and its mutated counterparts showed the highest Sn/Sp (92.6%/69.1% and 97.3%/92.3% for tiamulin and valnemulin, respectively). We identified the potential inactivation of the *tva*(A) protein via silent mutations and alternative codon usage, rendering a strain again susceptible to pleuromutilin antimicrobials (47). Including this information to assess predictive power allowed us to achieve the highest Sn/Sp for both pleuromutilins. This suggests that a sequencing-based approach might be preferable to a targeted PCR approach, allowing the identification of the aforementioned silent mutations. For tiamulin, the Sn could still be elevated to 99.1% by including the N148S mutation in the 50S ribosomal L3 protein, with a small decrease in Sp (66.7%).

Similarly, prediction of acquired lincomycin and tylvalosin resistance showed high Sn/Sp (91.8%/100% and 98.5%/92.9% for lincomycin and tylvalosin, respectively) when the A2153T (position 2058 per E. coli numbering) rather than the G846A (position 748 per E. coli numbering) mutation in the 23S rRNA gene binding domain was used (1, 48). Sensitivity to lincomycin could be further increased (93.8%) by including the presence of the transposon-associated *lnuC* gene (25). Interestingly, the gene was detected in only 6 strains, belonging to two distinct STs (ST132 and ST138). ST138 was shown to be closely linked to ST83, in which the *lnuC* gene was first described (Fig. 1) (25). Whether the presence of the *lnuC* gene within ST132 is the result of an independent transposition should be further elucidated.

Finally, acquired resistance to doxycycline was assessed using the genetic marker within the 16S rRNA gene (G1026C [position 1058 per E. coli numbering]). The Sn/Sp of this marker was promising, 92.5%/87.3%. While most genetic markers resulted in a high Sn (92% to 99%), some showed a lowered Sp (67% to 100%). This might be the result of setting phenotypic reference cutoffs that were too stringent, sacrificing Sp at the cost of Sn (49). Between the Sp values of our Belgian strains (Table S3A) and those from the UK (Table S3B) (1), a clear drop can be observed. This suggests once again the importance of the implementation of more robust AST phenotyping using a broth microdilution approach (14). Also, other resistance mechanisms or combinations of mechanisms might be involved, making it more difficult to link genetic and phenotypic AST.

It is important to note that here that the phenotypic approaches were used as a true reference. Nevertheless, also these approaches have their limitations. They are highly dependent on the successful growth of the B. hyodysenteriae strain but also the characteristics and stability of each antimicrobial. This was shown to hamper determination of ECOFF values for some antimicrobials in various other species, including Mycoplasma bovis (50, 51) and Mycoplasma hyopneumoniae (52). Next to genomic mutations and the presence/absence of genes, we suggested that the potential effect of silent mutations at the protein level [*tva*(A) protein] should be considered and taken into account in the assessment of acquired resistance. This adds an additional layer to the complexity of acquired resistance, in which other potential mechanisms (e.g., rRNA mutations) should also be considered to contribute to the observed phenotypes (29, 47). With regard to including genetic AST, one should keep in mind that various single nucleotide polymorphisms (SNPs) might not have any functional link with the observed phenotypes, as they are solely evolutionary traces. Most of these tend to be linked to specific STs, as was shown for the 16S and 23S rRNA genes (Fig. S1) in B. hyodysenteriae.

In conclusion, we showed the value of using long-read-only B. hyodysenteriae whole-genome sequencing to study epidemiology and assess the predictive power (Sn/Sp) of known and potential new genetic resistance markers in genetic AST for diagnostics in veterinary medicine. The presence of multidrug-resistant B. hyodysenteriae strains in Belgium is worrying and should be addressed with measures to fine-tune and speed up SD diagnostics, including AST. This would support practitioners in choosing targeted rather than empirical treatments. Our data showed good Sn (92% to 99%) for all tested antimicrobials, though Sp was lower for some of the tested antimicrobials (67% to 100%). In addition, our literature review on recent Belgian B. hyodysenteriae isolates suggests a re-evaluation of current cutoff values. A small ray of light can be found in the appearance of new STs with low to no acquired resistance to any of the approved antimicrobial drugs. Altogether, these data will contribute to the further development of quick and cost-efficient all-in-one diagnostics to determine the proper use of antimicrobials in veterinary medicine.

## MATERIALS AND METHODS

### Collection and growth of B. hyodysenteriae isolates.

A total of 92 B. hyodysenteriae isolates, originating from a bacterial collection from Belgian farms showing SD-like signs, were obtained between 2018 and 2020. Isolates originated from farms in nearly all provinces of Belgium: West Flanders (*n* = 66), East Flanders (*n* = 9), Antwerp (*n* = 7), Hainaut (*n* = 7), Limburg (*n* = 1), Flemish Brabant (*n* = 1), and Luxembourg (*n* = 1), of which West-Flanders is the area with the highest pig density (Fig. 1, inset). Obtaining B. hyodysenteriae cultures was performed as described by Jenkinson and Wingar and by Mahu et al., with the addition of rifampin (12.5 μg mL^−1^) and spiramycin (25 μg mL^−1^) to the growth media (53, 54). Next, all strains were phenotypically and genetically characterized starting from a frozen culture stock aliquot after inoculation on Columbia blood agar plates. The American B. hyodysenteriae type strain, B-78 (ATCC 27164), served as a positive control in all experiments. Most isolates originated from nonrelated farms, except for some strains. An overview of all samples, isolation dates, locations, and their relatedness is shown in Table S1.

### Phenotypic characterization of antimicrobial susceptibility in B. hyodysenteriae isolates.

For phenotypic evaluation of antimicrobial susceptibility, five antimicrobial compounds were included in this study. The compounds were selected based on their antibiotic class (lincosamide, macrolide, tetracycline, and pleuromutilin) and relevance to the field. All strains were tested at the following concentrations: lincomycin, 0.25 to 64 μg mL^−1^; tylvalosin, 0.03 to 128 μg mL^−1^; doxycycline, 0.125 to 16 μg mL^−1^; tiamulin, 0.03 to 16 μg mL^−1^; and valnemulin, 0.03 to 16 μg mL^−1^. MICs of all antimicrobials were determined using the agar dilution method as described by Vyt and Hommez (55). In each experiment, the B. hyodysenteriae type strain B-78 (ATCC 27164) was included as a quality control of the AST experiment. As described by Vyt and Hommez and by Pringle et al., MICs of all tested antimicrobial compounds should be in quality control ranges for this strain (55, 56). Evaluation of resistance was based on most recently published ECOFFs and CBs, respectively, as summarized by Stubberfield et al. (1): tiamulin, >0.25 and >2 μg mL^−1^; valnemulin, >0.125 μg mL^−1^; lincomycin, >1 and >16 μg mL^−1^; tylvalosin, >1 and >16 μg mL^−1^; and doxycycline, >0.5 and >4 μg mL^−1^. In addition, MIC_50_s and MIC_90_s were determined as the MICs that inhibited the growth of 50% and 90% of all tested isolates, respectively. Acquired multidrug resistance was reported if a strain showed resistance to one pleuromutilin (tiamulin or valnemulin), doxycycline, and one of the macrolide-lincosamide-streptogramin B (MLSB) antibiotics (lincomycin or tylvalosin). Some strains were excluded from further analyses, including those that were genetically classified as B. murdochii (*n* = 2), showed a mixed genotype (*n* = 2), or could not be recovered properly after storage (*n* = 1). MIC survival analyses were performed to visually compare MICs between different countries and times. Metadata (MICs) were extracted from the literature and imported into GraphPad Prism (v9.4.1) to generate survival curves.

### Extraction of high-molecular-weight DNA for long- and short-read sequencing.

All culturable B. hyodysenteriae strains (*n* = 86) were inoculated on Columbia blood agar plates and incubated for 3 to 7 days anaerobically at 37°C. All colonies from a fully grown plate were collected in 250 μL Dulbecco's phosphate-buffered saline (dPBS) (Gibco) and subjected to high-molecular-weight (HMW)-DNA isolation using the ZymoBIOMICS DNA miniprep kit (Zymo Research). The manufacturer’s instructions were followed, with the addition of a facultative proteinase K treatment (500 ng μL^−1^; 30 min at 55°C; Promega) after bead beating. The quantity and quality of the resulting HMW DNA was verified on a NanoDrop spectrophotometer, and samples with lower quality (*A*_260_/*A*_230_ < 1.7) were subjected to an additional cleanup. The latter was done using CleanNGS beads (CleanNA) in a 1:1 ratio, including a double 70% ethanol wash, a 5-min drying step at 50°C, and elution in 25 μL DNase/RNase-free water. High-quality HMW DNA was subsequently used in a rapid long-read sequencing library preparation (SQK-RBK-004; ONT) using 400 ng nonamplified DNA input per sample. Batches of 10 samples were multiplexed for nanopore sequencing on R9.4.1 flow cells for 48 h each, allowing the collection of raw fast5 files in the MinKNOW software (ONT). On each run, the B. hyodysenteriae type strain B-78 (ATCC 27164) was included. For 10 strains, short-read Illumina sequencing was also performed at MacroGen (South Korea), where a Nextera DNA XT library (Illumina) was constructed for paired-end sequencing on a NovaSeq 6000 system (Illumina).

### Training and evaluation of a B. hyodysenteriae specific Bonito base-calling model.

To construct complete and high-quality B. hyodysenteriae genomes, a custom-trained Bonito base-calling model was generated and validated as previously described by Vereecke et al. (21). First, raw Illumina reads were quality filtered and adapter trimmed using Trimmomatic (v.0.39) (57), specifying ILLUMINACLIP:NexteraPE-PE.fa:2:30:10 LEADING:3 TRAILING:3 MINLEN:45 options. Subsequently, adapter-trimmed reads were used to generate 10 B. hyodysenteriae *de novo* reference genomes in Unicycler (v. 0.4.9b) (58). Raw nanopore data (fast5) of the same 10 strains were base-called using Bonito (v.0.4.0; ONT), specifying basecaller dna_r9.4.1 –save-ctc –reference, which allowed linking raw squiggles of each strain with its corresponding Illumina reference sequence. In addition, saved CTC data files were combined and used for subsequent Bonito model training using the train –pretrained dna_r9.4.1@v3.3 –directory OUT_DIR –epochs 75 –chunks 1000000 –batch 128 command. All Bonito commands were run on a single node of the Ghent University High Performance Computer Tier 2 (Flemish Supercomputer Center) with 4× NVIDIA Volta V100 graphics processing units (GPUs; 32-GB GPU memory each) to speed up the training process. Performance of the new Bonito base-calling model was verified using consensus accuracy metrics (Pomoxis v.0.3.2; ONT), genome completeness (CheckM v.1.1.0) (59), and predicted genes (Quast v.5.0.2) (60).

### Generation of complete and high-quality B. hyodysenteriae genomes.

Raw nanopore sequencing data (fast5) of all strains (*n* = 86) were base-called using the custom-trained B. hyodysenteriae Bonito model on the Ghent University HPC Tier 2 GPU cluster Joltik using 1× GPU. Resulting FASTA files were reformatted to FASTQ files using the reformat.sh script (BBtools [B. Bushnell]; sourceforge.net/projects/bbmap/) giving an artificial Q score of 12 to each base. Next, complete and high-quality genomes were generated using an in-house *de novo* genome assembly pipeline. In short, draft genomes were assembled using canu (v.2.0) (61), followed by read mapping and polishing using minimap2 (v.2.20) (62) and medaka (v.1.5.0; ONT), respectively. Quality evaluation of final consensus genomes was done using Kraken2 (63), ribosomal MLST (64), and CheckM (v.1.1.0) (59). For the latter, completeness was evaluated against the 458 *Brachyspira* sp. marker sets (10 genomes with 783 marker genes). A completeness of 100% indicates that all 783 genes were identified successfully.

### Downstream genomic analyses for phylogeny, MLST, AMR, and virulence mediators and predictive value statistics.

Based on contamination and genome completeness, 81 of 86 B. hyodysenteriae genomes were considered for use in downstream analyses, including (i) MLST, (ii) phylogenetic inference, and (iii) a search for AMR mediators. First, MLST typing was performed on all strains through the online PubMLST platform (65). The B. hyodysenteriae-specific MLST scheme (including the *adh*, *alp*, *est*, *gdh*, *glpK*, *pgm*, and *thi* genes) was used as described by Råsbäck et al. (66). Sequence type profiles of all available B. hyodysenteriae strains were downloaded from PubMLST (*n* = 567) and manually supplemented with 115 profiles of UK strains (1, 11). A goeBURST full MLST analysis was performed in Phyloviz2 (v.2.0a) (67) as previously described by Feil et al. (68). Next, phylogenetic inference was drawn from the whole genomes using the SNP alignment from the csi phylogeny workflow (69), followed by maximum-likelihood (ML) tree construction in IQ-tree2 (-BB 1000) (70). This tree included one of the long-read-sequenced *de novo*-assembled B. hyodysenteriae B-78 strain as a reference. Also, all available and complete NCBI B. hyodysenteriae genomes (*n* = 56; accessed 7 September 2022) were included for phylogenetic inference, together with 154 genome assemblies that were reconstructed from raw data files from references 1, 11, and 37 using Trimmomatic (ILLUMINACLIP:TruSeq3-PE2.fa:2:30:10:2:TrueLEADING:3 TRAILING:3 MINLEN:36; v.0.39) (57) and SPAdes (v.3.13.1) (71). Tree visualizations were done using Interactive Tree Of Life (iTOL v.5) (72). Also, genomes were screened for AMR mediators using Abricate (v.1.0.1) (https://github.com/tseemann/abricate) and the CARD database (73). To address point mutations in 16S and 23S rRNA genes and associated ribosomal proteins, sequences were extracted from the genomes and subjected to a SNP analysis. Models for 16S and 23S rRNA structures were downloaded from http://www.rna.icmb.utexas.edu. To assess functionality of the *tva*(A) gene, a protein structure based on the reference sequence (LT970863) (74) was generated using Alphafold2 (75). Evaluation of rare codon usage in mutated *tva*(A) genes was done using the %MinMax Codon calculator as described by Clarke and Clark (76) and available at www.codons.org/index.html. A B. hyodysenteriae-specific codon usage table was included based on 62 coding sequences (20,177 codons) (www.kazusa.or.jp/codon/). Predictive values of genetic hallmarks for the observed acquired resistance phenotypes was determined as previously described by Trevethan (49). These predictive values were determined based on our 81 new Belgian isolates, supplemented with genotypes and phenotypes of 82 strains from the United Kingdom (1).

### Data availability.

All genomes were submitted to NCBI; the accession numbers for new sequences are presented in Table S1.
