# Supplementary material for: Predictive Power of Long-Read Whole-Genome Sequencing for Rapid Diagnostics of Multidrug-Resistant Brachyspira hyodysenteriae Strains
Source: Microbiol Spectr. 2023 Jan 5;11(1):e04123-22. doi: 10.1128/spectrum.04123-22 (PMC9927316; doi:10.1128/spectrum.04123-22)
Supplement: Supplemental file 2 — Tables S1-S4. Download spectrum.04123-22-s0002.pdf, PDF file, 0.24 MB [file spectrum.04123-22-s0002.pdf]

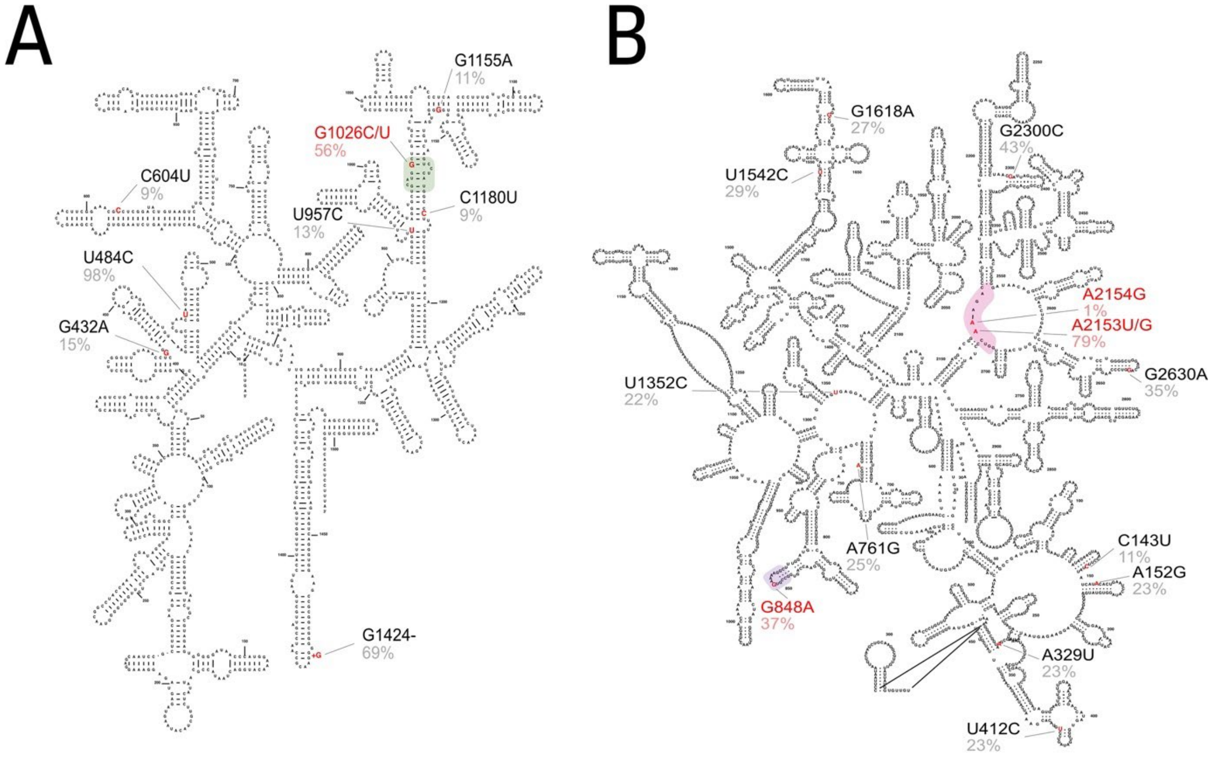


**Figure S1. Genetic hallmarks of lincosamides, macrolides, and tetracyclines in 23S and 23S rRNA model structures.** (A) Two-dimensional structure of 23S rRNA (URS00009CE9CC_1266923), highlighting the most abundant (>10%) SNPs within the studied population. Mutations previously associated with acquired resistance, are highlighted in red. Lincomycin alone and lincomycin/tylvalosin drug target regions, are shaded in purple and pink, respectively; (B) Two-dimensional structure of 16S rRNA (URS0000A194F0_1266923), highlighting the most abundant (>10%) SNPs within the studied population. Mutations previously associated with acquired resistance, are highlighted in red. Doxycycline drug target region is shaded in green.
